# Non-invasive prediction of detrusor underactivity in benign prostatic hyperplasia: an interpretable machine learning framework to optimize surgical selection

**DOI:** 10.3389/fmed.2026.1835415

**Published:** 2026-05-01

**Authors:** Long Gao, Zeming Luo, Yang Yuan, Zhen Luo, Hao Zhuang, Jianyong Gao, Xiaoshuang Xie

**Affiliations:** 1 Department of Urinary Surgery, Panzhihua Central Hospital, Panzhihua, China; 2 Department of Cardiovascular Medicine, Panzhihua Central Hospital, Panzhihua, China

**Keywords:** benign prostatic hyperplasia, detrusor underactivity, machine learning, SHAP analysis, XGBoost

## Abstract

**Objective:**

To develop and internally validate an interpretable, non-invasive machine learning framework to predict detrusor underactivity (DU) in patients with benign prostatic hyperplasia (BPH).

**Methods:**

This retrospective cohort study enrolled 538 urodynamically evaluated BPH patients. A rigorous multidimensional feature selection pipeline (LASSO, Boruta, and Recursive Feature Elimination) distilled 15 baseline clinical, anatomical, and uroflowmetry parameters into a parsimonious five-feature subset. Five supervised machine learning algorithms were trained and systematically compared. Shapley Additive exPlanations (SHAP) analysis was integrated for global and local interpretability.

**Results:**

The optimized XGBoost model demonstrated superior discriminatory performance (AUC = 0.958), significantly outperforming traditional multivariable logistic regression (AUC = 0.787). XGBoost consistently exhibited superior calibration and higher net clinical benefit across varied threshold probabilities. Crucially, SHAP global dependence plots revealed non-linear pathological trajectories, notably demonstrating a U-shaped risk profile for bladder wall thickness (BWT) that was not captured by classical linear statistical detection. Local SHAP visualizations effectively translated complex probabilistic outputs into individualized clinical reasoning.

**Conclusion:**

The interpretable XGBoost framework serves as a robust non-invasive risk stratification tool for DU, decoding complex non-linear clinical interactions. This algorithm holds significant potential to optimize preoperative patient selection and mitigate surgical failures in borderline clinical scenarios.

**Clinical trial registration:**

Identifier 2026-048.

## 1 Introduction

Benign prostatic hyperplasia (BPH) is highly prevalent among aging men, frequently manifesting as lower urinary tract symptoms (LUTS) secondary to bladder outlet obstruction (BOO) (1–3). Even though surgical treatment (e.g., transurethral resection of the prostate (4)) generally produces favorable outcomes in the management of obstructive BPH, a significant percentage of patients may experience persistent post-procedure voiding dysfunction (4, 5). Emerging clinical evidence attributes this suboptimal surgical efficacy largely to the unrecognized comorbid presence of detrusor underactivity (DU) (6, 7). DU is characterized by weakened and shortened detrusor contraction periods, which essentially impairs bladder emptying capacity despite anatomical de-obstruction (8, 9). Effective preoperative diagnosis of DU, therefore, is a critical factor in the optimization of surgical selection and the avoidance of unnecessary surgeries (10, 11).

Currently, the invasive urodynamic study (UDS) that includes pressure-flow analysis is the gold standard for diagnosing DU (12, 13). However, its widespread clinical implementation is encumbered by high costs, specialized equipment requirements, and patient discomfort as well as the possibility of iatrogenic infections of the urinary tract (14, 15). These practical limitations necessitate the development of robust, non-invasive predictive modalities based on the available clinical measures, like, transrectal ultrasonography (TRUS) measures and uroflowmetry (16). Although there have been extensive investigations, conventional univariable analyses and standard multivariable logistic regression models often provide contradictory diagnostic accuracies (17). This lack of consistency largely arises because of the profound symptomatic and functional overlap between isolated BOO and combined BOO with DU, wherein standard linear statistical assumptions fail to capture the complex and non-linear pathological remodelling of the bladder wall and prostate architecture (18).

Machine learning (ML) algorithms, including both linear and advanced tree-based ensembles, have substantial utility in identifying multi-dimensional, non-linear interactions in heterogeneous biomedical data sets (19). In contrast to classical regression methods, advanced ML models have the ability to automatically discover implicit thresholds and synergistic pathological features without arbitrary *a priori* structural constraints (20). However, the clinical translation of high-performance ML models is often hindered by their “black-box” nature, which lacks transparency regarding individual predictive reasoning (21). The recent integration of Shapley Additive exPlanations (SHAP) provides a rigorous mathematical framework to compute the marginal contribution of each feature, thereby providing global and local interpretability to complex algorithms (22).

Thus, the study was focused at constructing and internally validating an interpretable, non-invasive ML predictive framework of DU in patients with BPH. We conducted a systematic evaluation and comparison of the discriminative strength of various distinct ML algorithms by rigorously reducing comprehensive clinical, anatomical, and functional parameters down to a parsimonious feature set. By such a comparative method, we aimed to determine the most predictive model and to discover the non-linear underlying pathological trajectories underlying individualized clinical risk assessment by SHAP visualization.

## 2 Materials and methods

### 2.1 Study design and ethical approval

This retrospective, single-center cohort study was conducted following the ethical principles outlined in the Declaration of Helsinki. The study protocol was reviewed and approved by the Institutional Review Board (IRB) and the Ethics Committee of Panzhihua Central Hospital (Approval Number: 2026–048). Given the retrospective nature of the study, which utilized anonymized historical medical records without impacting patient care, the requirement for obtaining written informed consent was formally waived by the Ethics Committee.

### 2.2 Patient selection and data collection

We retrospectively analyzed a consecutive cohort of adult male patients with lower urinary tract symptoms (LUTS) resulting from benign prostatic hyperplasia (BPH) who had undergone a full clinical assessment and urodynamic studies (UDS). The diagnosis of detrusor underactivity (DU) was strictly established through pressure-flow studies during UDS, which served as the definitive reference standard. Patients with a history of neurogenic bladder dysfunction, prior lower urinary tract surgeries, prostate cancer, active urinary tract infections, or incomplete medical records were systematically excluded.

Comprehensive baseline demographic and clinical parameters were retrieved from electronic health records. The initial feature set comprised 15 variables, encompassing age, body mass index (BMI), duration of symptoms, total prostate-specific antigen (tPSA), free-to-total PSA ratio, transrectal ultrasonography (TRUS) anatomical metrics (total prostate volume [TPV], transitional zone volume [TZV], transitional zone index [TZI], intravesical prostatic protrusion [IPP], bladder wall thickness [BWT]), non-invasive uroflowmetry parameters (maximum flow rate [Qmax], post-void residual [PVR]), and subjective symptom evaluations (International Prostate Symptom Score [IPSS]-Voiding, IPSS-Storage, and Quality of Life [QoL] scores).

### 2.3 Feature engineering and dimensionality reduction

A rigorous multidimensional feature selection pipeline was implemented to mitigate the curse of dimensionality, reduce multicollinearity, and enhance model generalizability. To strictly prevent data leakage, the entire multidimensional feature selection pipeline was executed exclusively within the training dataset following the train-test split. Initially, Least Absolute Shrinkage and Selection Operator (LASSO) regression with 10-fold cross-validation was applied to the training data to shrink redundant variable coefficients. Subsequently, within the same training subset, the Boruta algorithm, a permutation-based random forest wrapper, was utilized to statistically categorize features into three tiers of feature importance as Confirmed, Tentative, and Rejected. Recursive Feature Elimination (RFE) was further employed to corroborate the optimal subset dimension across five-fold cross-validation. Finally, to ensure the statistical independence of the selected predictors, Variance Inflation Factors (VIF) were calculated, and a strict criterion of VIF < 5 was determined to decisively rule out the presence of serious multicollinearity.

### 2.4 Machine learning model development and interpretability

The preprocessed dataset was partitioned into a training set (70%) and a testing set (30%) using a stratified random sampling approach based on detrusor underactivity status to ensure the target variable was proportionately represented in both cohorts. A detailed comparison demonstrating the statistical comparability of baseline characteristics between the training and testing sets is provided in Supplementary Table S1. To determine the optimal model, five different supervised machine learning methods were systematically trained and compared: extreme gradient boosting (XGBoost), Random Forest (RF), Support Vector Machine (SVM), K-Nearest Neighbors (KNN) and the classic multivariate Logistic Regression (LR) model. Model hyperparameters were optimized using an exhaustive GridSearch strategy with an internal 5-fold cross-validation within the training cohort. For the XGBoost model, the specified search space included: learning rate (0.01, 0.05, 0.1), max depth (3, 5, 7), number of estimators (100, 300, 500), and subsample (0.8, 1.0). The final optimal hyperparameters selected were a learning rate of 0.05, max depth of 5, 300 estimators, and a subsample rate of 0.8.

The discriminatory performance of each algorithm on the independent testing set was evaluated using Area Under the Receiver Operating Characteristic Curve (AUC-ROC), Precision-Recall (PR) curves, accuracy, sensitivity, specificity, and Precision. To evaluate the agreement between the predicted probabilities and observed frequencies, calibration curves were plotted. In addition, Decision Curve Analysis (DCA) was conducted in order to measure the net clinical benefit of the best model at different threshold probabilities.

Shapley Additive exPlanations (SHAP) analysis was integrated into the optimized XGBoost model to overcome the inherent ‘black-box’ limitations of these advanced ensemble algorithms. Global and local interpretability (quantifying the overall feature significance and non-linear dependence curves) were produced to transform advanced mathematical output into clinically realistic results. R software (version 4.4. 1; R Foundation for Statistical Computing, Vienna, Austria) was used to perform all the statistical analyses and visualization. A *p*-value of less than 0.05 was found to be significant at the two-sided level.

## 3 Results

### 3.1 Exploratory data analysis and clinical feature distributions in BPH patients stratified by detrusor underactivity (DU) status

A total of 538 eligible patients with benign prostatic hyperplasia (BPH) were enrolled in this retrospective cohort and stratified into the Detrusor Underactivity (DU) group (n = 243) and the Non-DU group (n = 295) based on urodynamic findings. The baseline demographic and clinical characteristics are summarized in Table 1.

**Table 1:** Baseline characteristics of BPH patients stratified by Detrusor Underactivity status.

Variable	Overall (*n* = 538)	Non-DU (*n* = 295)	DU (*n* = 243)	*p*-value
Demographics and clinical history				
Age (years), Mean (SD)	69.83 (8.73)	69.42 (8.76)	70.34 (8.68)	0.224
BMI (kg/m ^2^), Mean (SD)	24.04 (3.08)	24.00 (3.10)	24.08 (3.05)	0.753
Duration of symptoms (months), Median [IQR]	38.00 [24.00, 55.00]	38.00 [23.00, 51.50]	40.00 [25.00, 58.00]	0.128
Laboratory parameters				
Total PSA (ng/mL), Median [IQR]	4.12 [3.53, 4.84]	4.19 [3.58, 4.90]	4.03 [3.48, 4.81]	0.314
f/t PSA ratio, Mean (SD)	0.21 (0.06)	0.21 (0.06)	0.21 (0.06)	0.875
Ultrasonography and urodynamic parameters				
Total prostate volume (TPV, mL), Median [IQR]	51.40 [41.12, 60.77]	51.40 [41.25, 60.75]	51.40 [41.05, 60.80]	0.890
Transitional zone volume (TZV, mL), Median [IQR]	25.20 [20.22, 30.58]	25.30 [20.20, 30.45]	25.00 [20.40, 30.90]	0.869
Transitional zone index (TZI), Mean (SD)	0.50 (0.06)	0.50 (0.06)	0.50 (0.06)	0.402
Intravesical prostatic protrusion (IPP, mm), Median [IQR]	7.20 [3.60, 11.30]	6.30 [3.30, 10.40]	8.40 [4.45, 12.30]	**<0.001**
Bladder wall thickness (BWT, mm), Mean (SD)	4.54 (1.72)	4.43 (1.34)	4.67 (2.08)	0.107
Post-void residual (PVR, mL), Median [IQR]	114.50 [67.00, 157.00]	90.00 [48.00, 137.50]	138.00 [102.00, 177.50]	**<0.001**
Maximum flow rate (Qmax, mL/s), Median [IQR]	8.85 [5.90, 11.90]	9.90 [7.35, 12.55]	7.40 [5.10, 10.95]	**<0.001**
Symptom scores				
IPSS-Voiding score, Mean (SD)	12.55 (4.32)	12.16 (4.47)	13.03 (4.09)	**0.020**
IPSS-Storage score, Mean (SD)	6.94 (2.78)	6.79 (2.77)	7.13 (2.78)	0.165
Quality of Life score (QoL), Mean (SD)	4.03 (1.24)	3.98 (1.25)	4.10 (1.22)	0.253

DU, Detrusor Underactivity; BMI, Body Mass Index; PSA, Prostate-Specific Antigen; IPSS, International Prostate Symptom Score; SD, Standard Deviation; IQR, Interquartile Range.

*p*-values were calculated using Student’s *t*-test for normally distributed continuous variables and the Mann–Whitney *U*-test for non-normally distributed variables. Significant *p*-values (<0.05) are indicated in bold.

**Figure 1:**
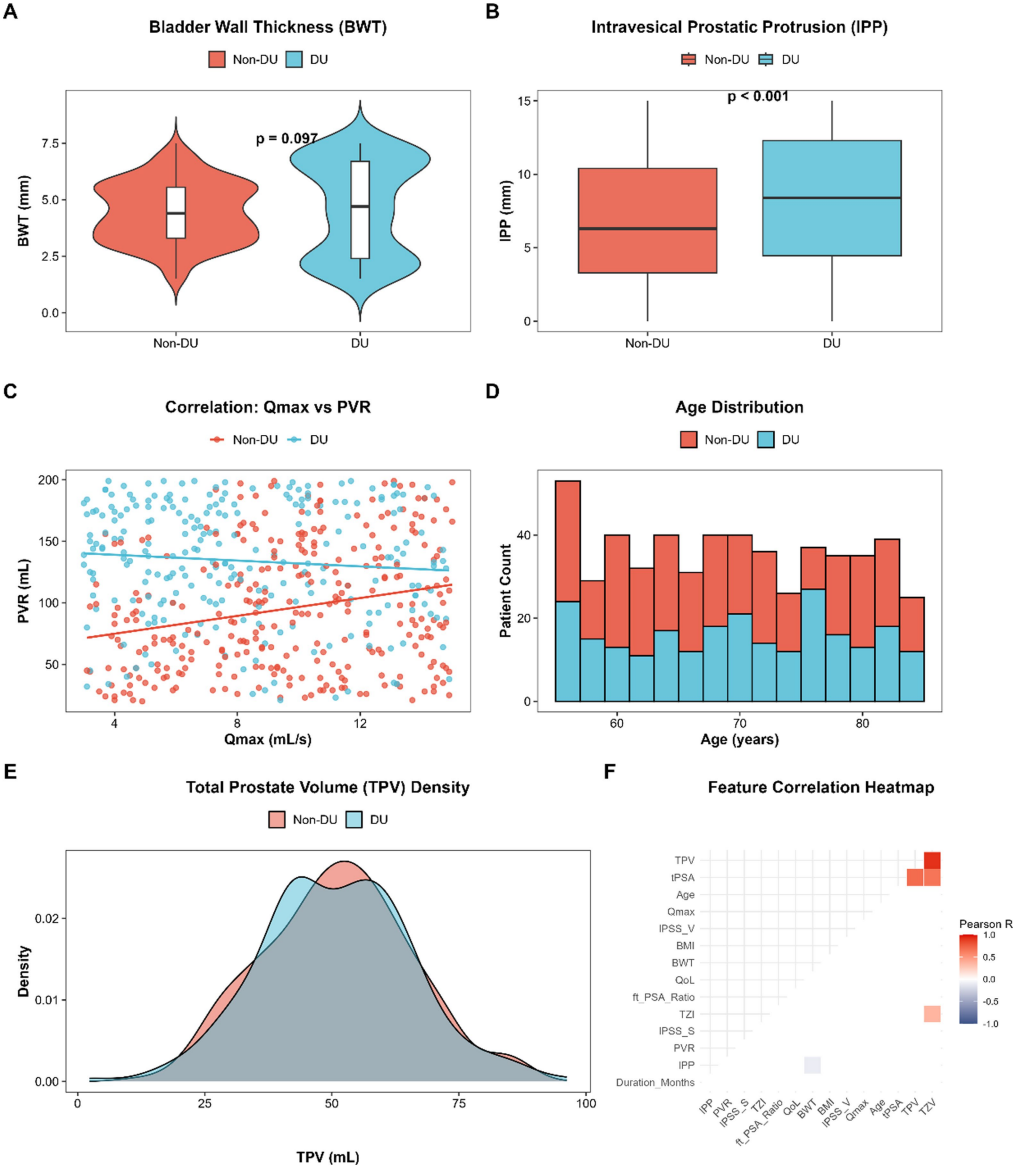
Exploratory data analysis and clinical feature distributions in benign prostatic hyperplasia (BPH) patients stratified by detrusor underactivity (DU) status. **(A)** Violin plot demonstrating the distribution of bladder wall thickness (BWT). **(B)** Box plot of intravesical prostatic protrusion (IPP). **(C)** Scatter plot illustrating the distribution of maximum flow rate (Qmax) and post-void residual (PVR) volumes across DU and non-DU groups. **(D)** Stacked histogram of patient age distribution. **(E)** Density plot of total prostate volume (TPV). **(F)** Pearson correlation heatmap of evaluated baseline variables.

Univariable analysis demonstrated high comparability between the two cohorts regarding demographic profiles and anatomical prostate parameters. Specifically, no statistically significant differences were observed in mean age (70.34 vs. 69.42 years, *p* = 0.224), which was further visualized by the highly comparable stacked age distribution (Figure 1D). Similarly, overall prostate size distributions exhibited high homogeneity (median TPV: 51.40 [IQR: 41.05–60.80] vs. 51.40 [IQR: 41.25–60.75] mL, *p* = 0.890) as corroborated by the largely overlapping density plots between the DU and Non-DU cohorts (Figure 1E). Also, the Pearson correlation analysis (Figure 1F) proved the lack of any strong multicollinearity between separate baseline clinical parameters except the anticipated anatomical derivations (e.g., TPV and TZV). This baseline homogeneity effectively minimized the potential confounding effects of general aging and gross prostatic enlargement on the primary outcome.

Conversely, substantial functional and anatomical differences were observed in certain clinical parameters. The median post-void residual volume (PVR: 138.00 vs. 90.00 mL, *p* < 0.001) and median maximum flow rate (Qmax: 7.40 vs. 9.90 mL/s, *p* < 0.001) of the DU patients were significantly higher and lower than that of the Non-DU patients. The DU cohort had a greater median intravesical prostate protrusion (IPP) anatomically, which was significantly greater (8.40 vs. 6.30 mm, p < 0.001, Figure 1B). However, as illustrated in the scatter plot (Figure 1C), substantial overlap existed between the two groups regarding Qmax and PVR, underscoring the diagnostic ambiguity when relying solely on conventional clinical thresholds.

Interestingly, the conventional statistical analysis showed that there was no significant difference between the mean of bladder wall thickness (BWT) between the DU and Non-DU groups (4.67 vs. 4.43 mm, *p* = 0.107). However, the descriptive analysis based on violin plots (Figure 1A) demonstrated that the distribution of BWT is highly polarized, exhibiting a bimodal-like pattern exclusively within the DU group (the standard deviation of BWT is significantly greater 2.08 vs. 1.34, interquartile range is also much greater 4.30 vs. 2.25). This complex structural variance—encompassing both extreme detrusor thinning and severe fibrotic thickening—suggested a profound non-linear pathological progression that evaded detection by standard linear comparative tests, providing an essential argument in support of the implementation of advanced machine learning algorithms.

### 3.2 Multidimensional feature selection and collinearity assessment

Since the structural variance, and non-linearities of the distributions were detected during the exploratory baseline analysis, a strict multidimensional feature selection approach was used to detect the strongest predictors of detrusor underactivity (DU), in order to prevent model overfitting.

**Figure 2:**
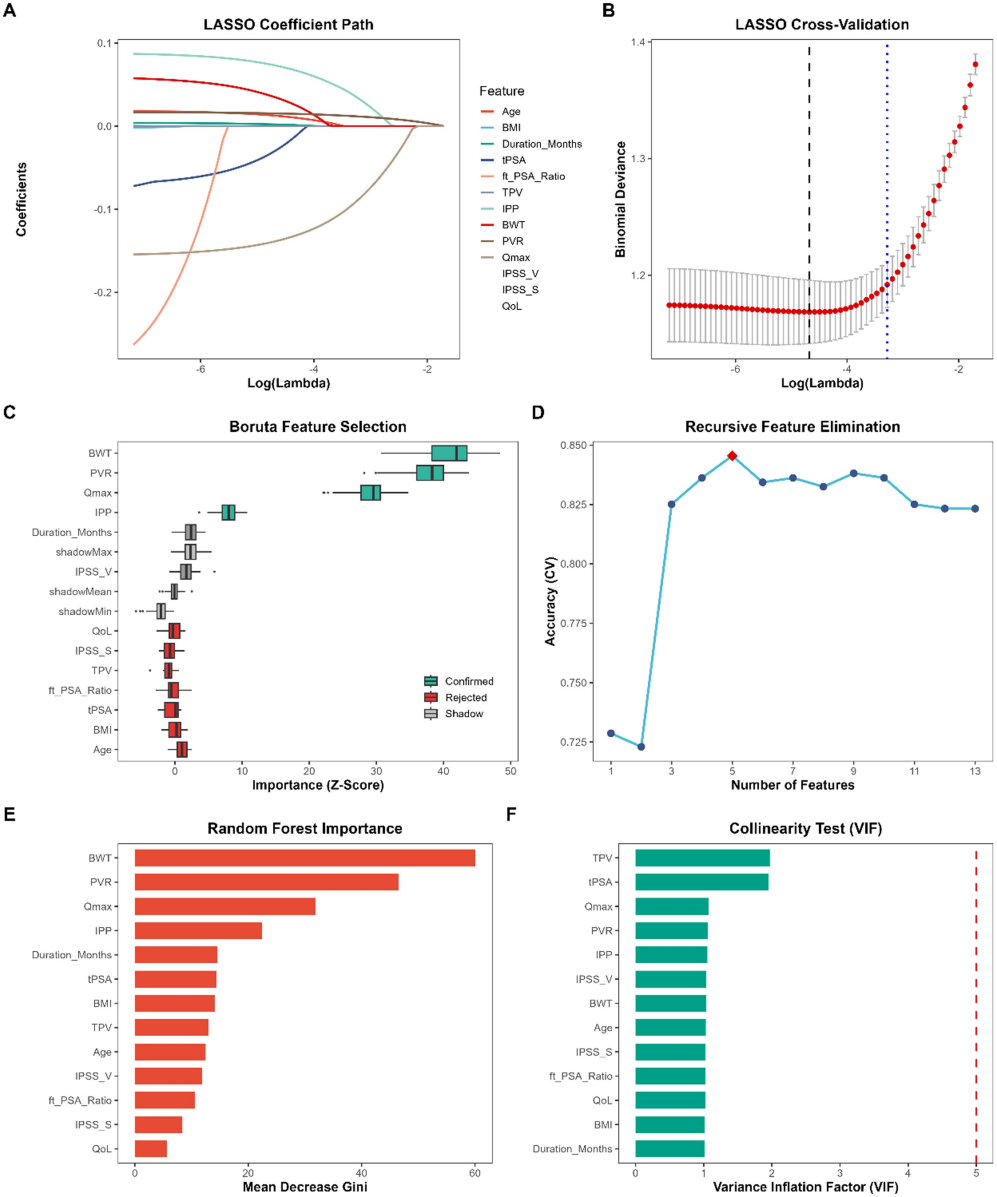
Multidimensional feature selection and collinearity assessment. **(A)** Least absolute shrinkage and selection operator (LASSO) coefficient profiles of the clinical features. **(B)** LASSO cross-validation plot displaying the binomial deviance against log(lambda). **(C)** Boruta feature selection plot ranking variables by *Z*-score, with green boxplots representing “Confirmed” predictors. **(D)** Recursive feature elimination (RFE) curve showing cross-validation accuracy across different subset sizes. **(E)** Feature importance ranked by mean decrease Gini in the random forest model. **(F)** Variance inflation factor (VIF) values for the final selected variables, with the red dashed line indicating the threshold of 5.

Firstly, LASSO regression using 10-fold cross-validation was applied to assess the coefficient paths and penalize redundant variables (Figures 2A,B). Permutation based feature selection algorithm, Boruta was employed to further identify highly relevant predictors. Four clinical variables, i.e., Confirmed (BWT, PVR, Qmax and IPP) were explicitly identified, whereas the symptom duration and IPSS-Voiding score were treated as Tentative, as can be seen in Figure 2C. It should be mentioned that the algorithm disregarded the concept of classical demographic and anatomical values, that is, the age of a patient and the total prostate volume (TPV). The direction of this trend is that foregoing predictive value of gross enlargement of the prostate gland and aging may be significantly mediated or overtaken by downstream functional abnormalities (e.g., alteration of urine flow) and localized detrusor morphological abnormalities.

Recursive Feature Elimination (RFE) was used to further validate the optimum dimension of the subset of features. The RFE learning curve (Figure 2D) demonstrated that the cross-validation accuracy peaked when exactly five core features were retained. This is the best subset size which was very similar with the internal feature rank ratings generated by the use of the Random Forest model (Figure 2E). According to the Mean Decrease Gini index, BWT, PVR, Qmax, IPP and duration of symptoms were the five most dominant predictors. The emergence of BWT as the most critical feature logically aligns with the pronounced distributional variance observed in our initial univariable analysis.

Lastly, a collinearity diagnostic test was conducted to ensure the statistical stability and interpretability of the ensuing machine learning models. As presented in Figure 2F, the Variance Inflation Factors (VIF) for all evaluated variables were well below the stringent threshold of 5, confirming the complete absence of severe multicollinearity among the selected clinical indicators. Accordingly, the five mutually independent and distant predictive features were included in the latter phase of building the final model.

### 3.3 Performance evaluation and comparison of machine learning models for predicting DU

Building upon the identification of the optimal five-feature subset, five distinct machine learning algorithms were trained and evaluated to predict the risk of detrusor underactivity (DU). Testing cohort was used to assess and compare the performance in terms of prediction.

**Figure 3:**
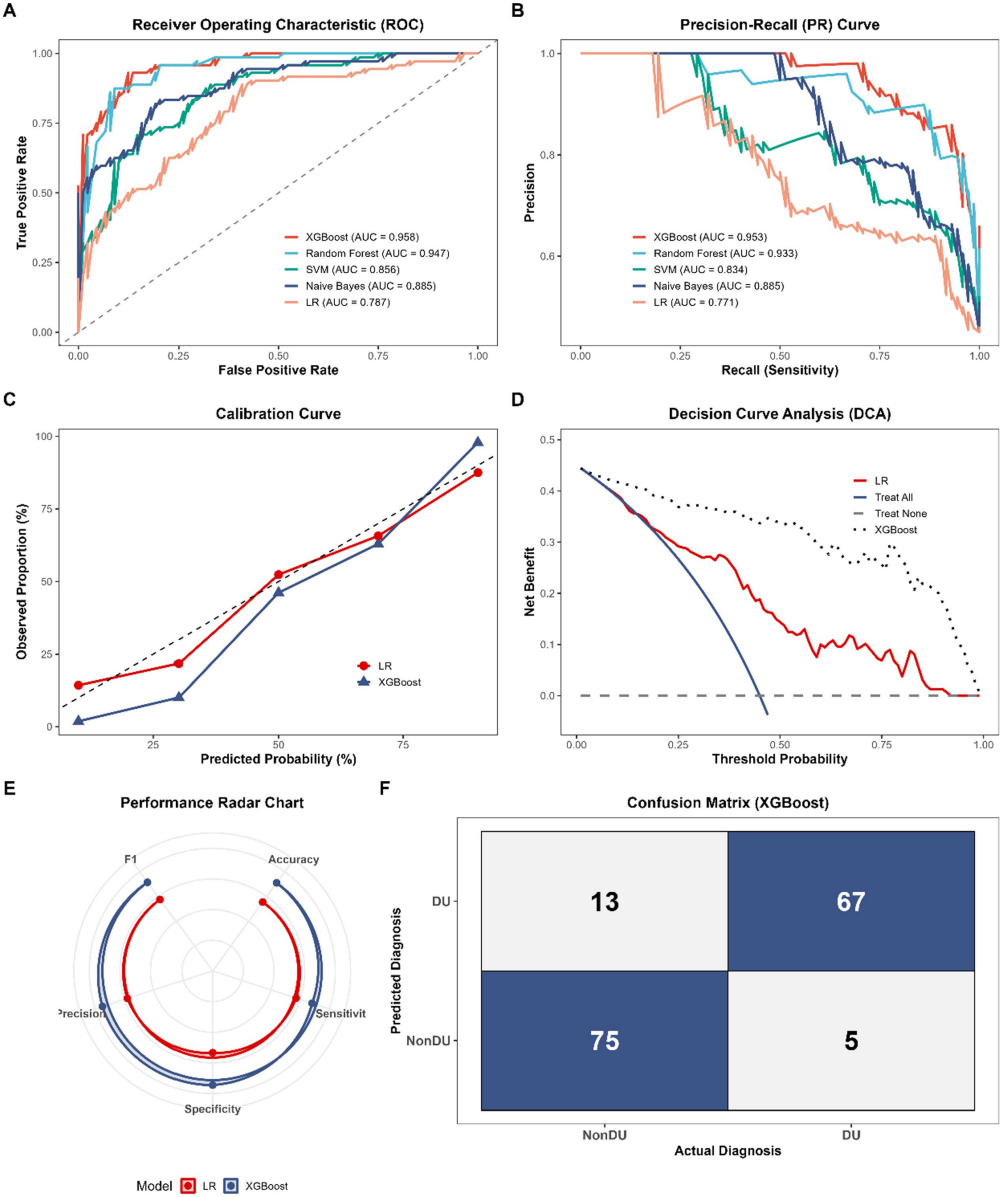
Performance evaluation and comparison of machine learning models for predicting DU. **(A)** Receiver operating characteristic (ROC) curves and corresponding area under the curve (AUC) values for the prediction models. **(B)** Precision-recall (PR) curves. **(C)** Calibration curves comparing predicted probabilities against observed frequencies. **(D)** Decision curve analysis (DCA) of the evaluated models alongside ‘Treat All’ and ‘Treat None’ baseline strategies. **(E)** Radar chart visualizing the multidimensional performance metrics (Accuracy, Sensitivity, Specificity, F1-score, and Precision) of the XGBoost and Logistic Regression models. **(F)** Confusion matrix of the optimized XGBoost model on the testing set.

The ability to discriminate was first visualised by Receiver Operating Characteristic (ROC) and Precision-Recall (PR) curves of each model. As illustrated in Figure 3A, the XGBoost model achieved the highest area under the ROC curve (AUC = 0.958, 95% CI: 0.925–0.991), substantially outperforming the traditional Logistic Regression (LR) model (AUC = 0.787, 95% CI: 0.712–0.862). This dominance was highly reflected by the Random Forest algorithm (AUC = 0.947), as it indicates that tree-based ensembles can effectively capture the non-linear pathological interaction as hypothesized in our baseline analysis. Likewise, when comparing the imbalanced clinical datasets, which were of particular interest in the XGBoost use, the PR curve (Figure 3B) also supported the fact that the method was the most precise and sensitive in terms of the area under the curve (0.953).

The probabilistic predictions were quantitatively and visually assessed using calibration curves, incorporating the Brier score, calibration slope, and intercept. Figure 3C shows that both XGBoost and LR models indicated a reasonable overall fit between the expected frequency and the observed ones. Nevertheless, XGBoost showed a superior overall alignment with the theoretical diagonal line. However, a slight deviation was observed at the extreme highest-risk prediction intervals, suggesting a minor tendency of the model to marginally underestimate risk in the most severe clinical presentations, though this does not significantly compromise its general clinical utility for identifying high-risk patients.

In order to further measure the multidimensional predictive measures, a performance radar mapping was prepared (Figure 3E) to compare optimized XGBoosts model and the baseline LR model. XGBoost demonstrated superior performance across all evaluated dimensions, notably in overall accuracy (0.875 vs. 0.694) and sensitivity (0.852 vs. 0.716), as well as specificity (0.903 vs. 0.667). XGBoost achieved an F1-score and a precision of 0.882 and 0.915 respectively, far exceeding those of the LR model (0.720 and 0.724). The robust classification capability of XGBoost is explicitly detailed in its confusion matrix (Figure 3F), recording a remarkably small false-negative count and correctly identifying 67 out of 72 actual (DU) cases in the testing subset.

To evaluate the practical value of these models, we applied Decision Curve Analysis (DCA) as shown in Figure 3D. Incorporating the XGBoost algorithm into the diagnostic workflow yielded a consistently higher net clinical benefit across nearly the entire range of threshold probabilities when compared to the traditional LR approach, as well as the “Treat All” (representing a strategy where all patients undergo invasive urodynamic studies) and “Treat None” (representing a strategy where no patients undergo invasive testing) baselines. Such a distinct advantage in net benefit highlights that relying on this XGBoost-driven framework could better guide personalized surgical decisions for BPH patients.

### 3.4 Global interpretability of the XGBoost model using SHAP analysis

**Figure 4:**
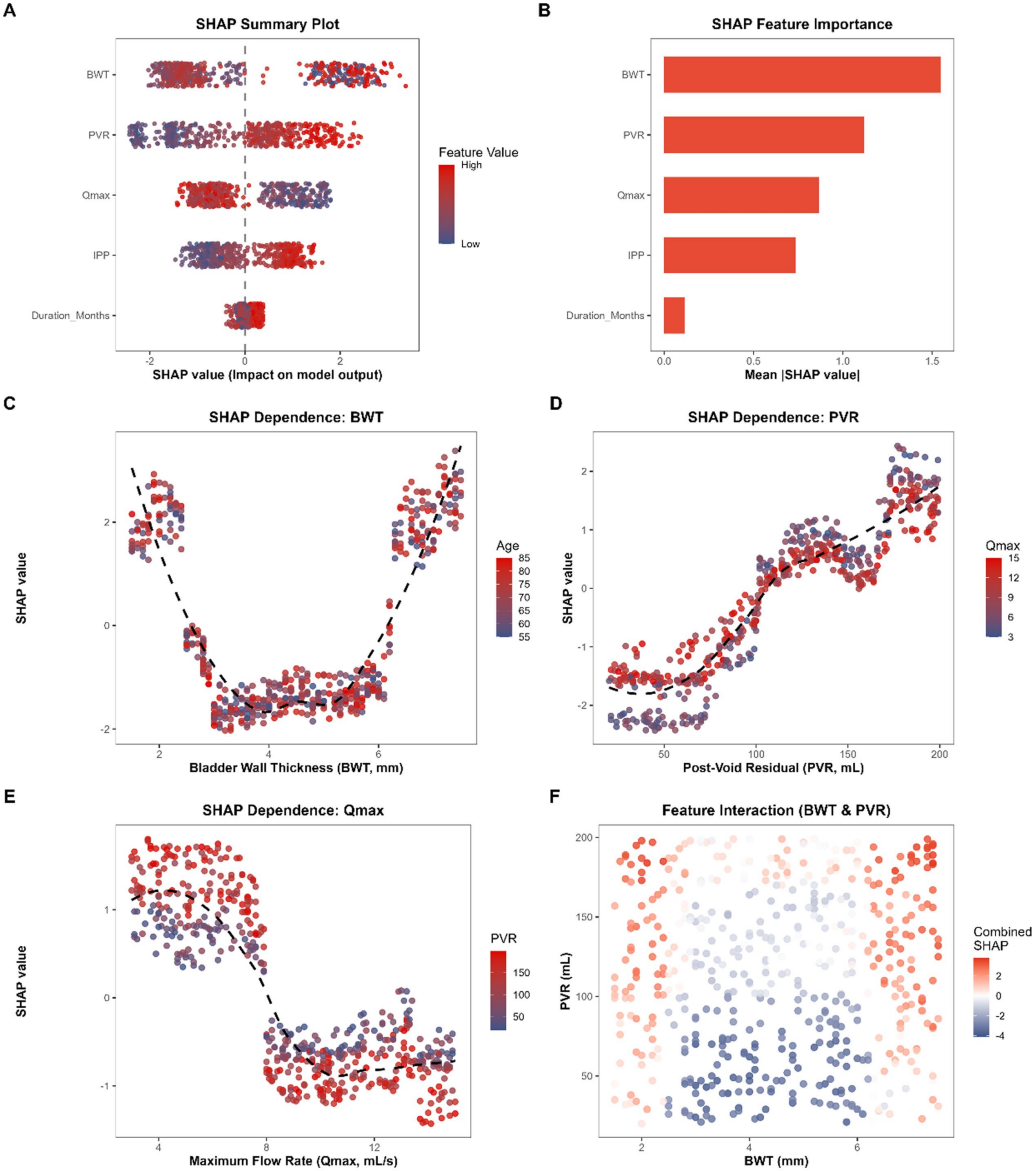
Global interpretability of the XGBoost model using Shapley additive explanations (SHAP) analysis. **(A)** SHAP summary plot illustrating the distribution of feature impacts on the model output, with colors representing original feature values. **(B)** SHAP feature importance ranking based on the mean absolute SHAP value. **(C–E)** SHAP dependence plots for bladder wall thickness (BWT) **(C)**, post-void residual (PVR) volume **(D)**, and maximum flow rate (Qmax) **(E)**. The overlaid black dashed lines represent locally estimated scatterplot smoothing (LOESS) curves. Color gradients denote the interacting features (Age, Qmax, and PVR, respectively). **(F)** Two-dimensional (2D) interaction scatter plot showing the combined SHAP values of BWT and PVR.

To demystify the ‘black-box’ nature of the optimized XGBoost algorithm and to elucidate the underlying pathophysiological mechanisms driving the predictions, Shapley Additive exPlanations (SHAP) global analysis was conducted. Macroscopic direction and strength of the effect of individual predictors on detrusor underactivity (DU) risks were first visualized with the help of SHAP summary plot (Figure 4A). In line with this, the importance of the features was represented by the mean absolute SHAP values (Figure 4B). Bladder wall thickness (BWT) emerged as the paramount determinant of DU risk (Mean |SHAP| = 1.547), next came post-void residual volume (PVR: 1.121) and maximum flow rate (Qmax: 0.865). The moderate but constant contributions were exerted by intravesical prostatic protrusion (IPP: 0.734) and duration of symptoms (0.115).

More importantly, the application of the Locally Estimated Scatterplot Smoothing (LOESS) curves on the SHAP dependence plots identified underlying non-linear continuous trends that were not inherently reflective in the basic linear model. Figure 4C, The SHAP dependence plot of BWT had an impressive virtually symmetrical U-shaped pathologically trending curve. Particularly, those BWT values which deviated out of the physiological range, either due to profound detrusor decompensation (extreme thinness, e.g., BWT < 3.0 mm) or due to severe fibrotic hypertrophy (excessive thickening, e.g., BWT > 6.0 mm), provoked the positive SHAP values to a much greater extent. On the other hand BWT in the intermediate range of compensatory (c. 4.0 mm) produced a strong protective or neutral effect. Such a complex bi-directional variance is a clear example to confirm our first hypothesis made in the baselines analysis (Table 1) that supports the absolute need to apply tree-based ensemble framework.

In addition, the classical threshold effects have been clearly reflected to the functional urodynamic parameters. The dependence curve of SHAP (PVR, Figure 4D) exhibited a sharp, sigmoidal-shaped increase in risk after the residual volume had greater than about 100 mL, and reached the plateau at exceptionally large volumes. Similar findings were noted regarding the inverse threshold of Qmax (Figure 4E), as the protective effect of the flow rate diminished and transitioned into a dominant risk factor at a flow rate that decreased to less than 8 mL/s. Interestingly, color mapping in Figures 4D,E revealed subtle interactive gradients, suggesting that the risk conferred by high PVR was further exacerbated when concurrently presenting with low Qmax (blue dots in the upper-right quadrant of Figure 4D).

In order to further describe these complex synergistic risks, a 2D SHAP feature interaction plot was developed on the two best predictors, BWT and PVR (Figure 4F). Combined SHAP heatmap demonstrated a clear distinction of a high-risk pathological zone (deep red dots) mainly centered on patients who had large remaining urine portions at the same time as they all had an extreme bladder wall change (either atrophic or hypertrophic). This visual inspection is a mathematical expression of the XGBoost outputs derived into visual, highly interpretable multidimensional clinical phenotypes.

### 3.5 Local individualized SHAP explanations and clinical case studies

In order to address the gap between the machine learning algorithmic outputs and realistic clinical reasoning, local SHAP explanations were created in order to break down the individual predictive paths of individual patients. That approach describes the fact that the complex interaction of the five predictors in focus adds to the final diagnostic potential.

**Figure 5:**
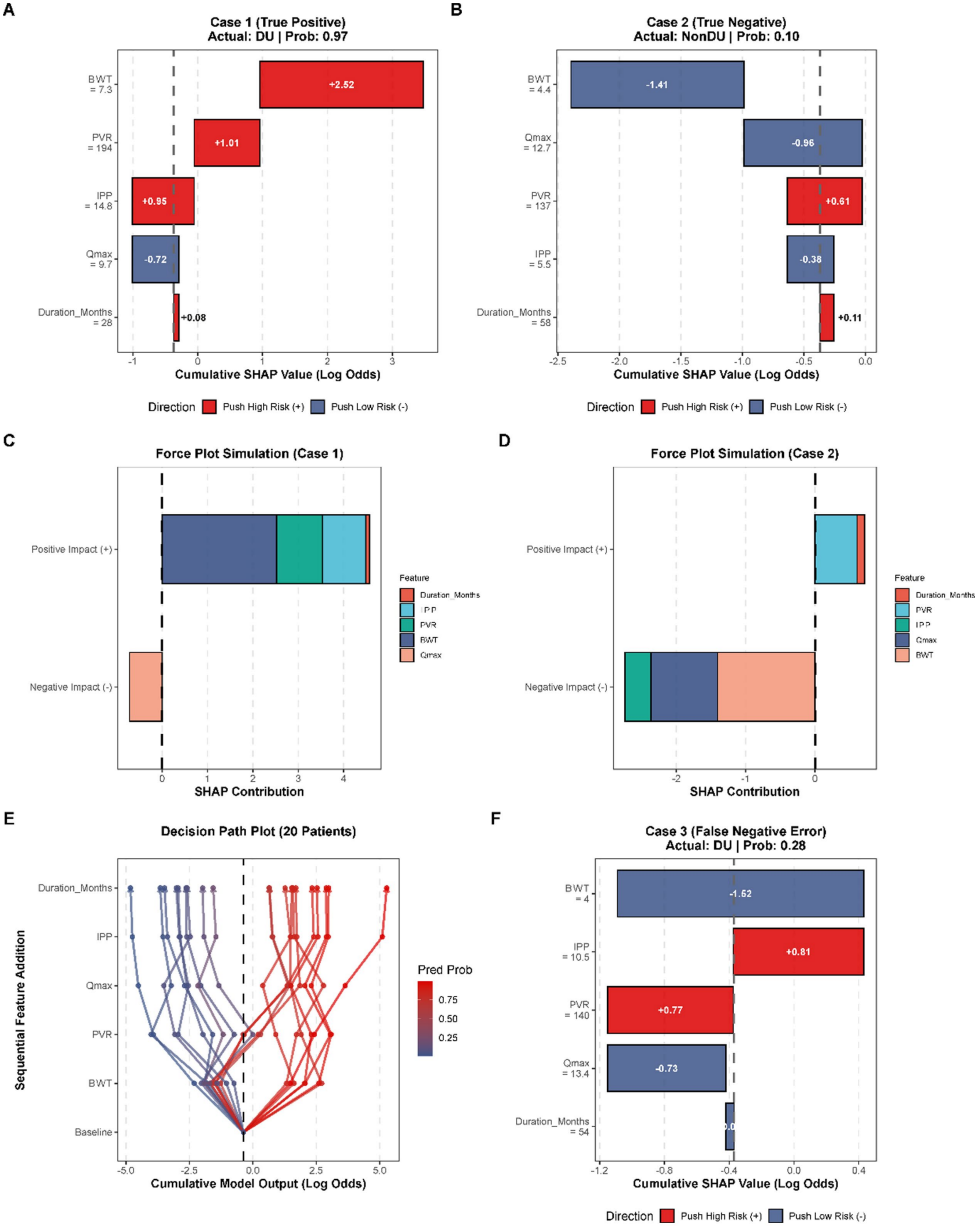
Local individualized Shapley additive explanations (SHAP) for specific clinical cases. **(A,B)** Waterfall plots illustrating the step-by-step accumulation of SHAP values for a true positive patient (Case 1) and a true negative patient (Case 2). **(C,D)** Force plots summarizing the magnitude and direction of feature impacts for Case 1 and Case 2. **(E)** Decision path plot tracking the sequential feature addition and cumulative log-odds output for 20 randomly selected patients. **(F)** Waterfall plot for a false negative patient (Case 3), showing the individual feature contributions to the predicted probability.

For a representative high-risk patient (Case 1, predictive probability: 0.97, Figure 5A), the waterfall plot precisely delineated the sequential accumulation of risk. The most substantial detrimental impact was propelled by highly thickened bladder wall (BWT = 7.3 mm), and yielded a substantial positive SHAP effect (+2.52 log odds) that aligned with the fibrotic hypertrophy pathology found in the global analysis (Figure 4C). This low-level protective action of an acceptable maximum flow rate (Qmax = 9.7 mL/s, SHAP: −0.72) outweighed by the compounding risks of an increased post-void residual volume (PVR = 194 mL, SHAP: +1.01) and a pronounced intra-vesical protrusion of the prostate (IPP = 14.8 mm, SHAP: +0.95). It was also possible to summarize the total magnitude of such opposing forces into the corresponding force plot simulation (Figure 5C).

The model, in its turn, was right in identifying a True Negative patient (Case 2, a predicted probability: 0.10, Figure 5B). This patient was deep into the low-risk category, despite the moderate elevating residual volume (PVR = 137 mL, with a contributory +0.61 to risk). The protective mechanism was predominantly orchestrated by a physiological thickness of the bladder wall (BWT = 4.4 mm, SHAP: −1.41) and a high urinary flow rate (Qmax = 12.7 mL/s, SHAP: −0.96). The resultant force plot (Figure 5D) indicated that the protective forces were sufficient to overcome the isolated risk factor hence indicating the hopefully well compensated detrusor activity.

To depict the risk divergence at the level of cohort, a 20 randomly chosen patients decision path plot (Figure 5E) was plotted. The divergent paths indicated that BWT and PVR were the pivotal bifurcating points, which aggressively divided the cohort of patients into high-risk (red paths) and low-risk (blue paths) groups. Lastly, a patient who was a False Negative patient (Case 3, actual DU but predicted probability: 0.28, Figure 5F), underwent the error analysis to critically test possible limitations of the model. Although the waterfall plot revealed that, despite the presence of a very severe anatomic obstruction (IPP = 10.5 mm, SHAP: +0.81) and residual urine (PVR = 140 mL/s, SHAP: +0.77), an apparent normal bladder wall thickness (BWT = 4.0 mm, SHAP: −1.52) and maximum flow rate (Qmax = 13.4 mL/s, SHAP: −0.73) greatly misrepresented the risk profile. The combination of these parameters produced disproportional protective SHAP forces that practically concealed the underlying detrusor failure. Such a misclassification helps to emphasize the complexity nature of masked clinical manifestations and implies that additional invasive urodynamic tests could be justified in border situations with paradoxical physiological values to prevent underdiagnosis.

## 4 Discussion

Accurate preoperative identification of detrusor underactivity (DU) in men with benign prostatic hyperplasia (BPH) remains a formidable clinical challenge due to the similarity between symptoms of a bladder outlet obstruction and those of a disordered detrusor contractility (23). Recent advancements in 2024 and 2025 have demonstrated the promising yet variable performance of artificial intelligence, particularly tree-based algorithms like XGBoost and CatBoost, in the non-invasive discrimination of lower urinary tract symptoms and detrusor underactivity (13, 24, 25). Building incrementally upon this evolving literature, our research developed and internally verified an interpretable, non-invasive XGBoost framework. This model successfully distilled 15 standard clinical parameters into a parsimonious five-feature subset, achieving robust diagnostic performance (AUC = 0.958) compared to traditional linear logistic regression (AUC = 0.787).

The non-linear predictive capability of bladder wall thickness (BWT) resulted in one of the key findings of our studies. Previous studies examining BWT as a surrogate endpoint that reflects the state of bladder dysfunction have largely provided conflicting data, as some researchers promoted the use of this method and others showed no significant correlation between the two (26, 27). This controversy was reflected in our baseline univariable analysis which found no statistically significant difference in the mean BWT of the DU and Non-DU cohorts (*p* = 0.107). However, by deploying SHAP global interpretability analysis, we uncovered a striking, bimodal-like U-shaped continuous pathological trajectory. This curve indicated that extreme detrusor thinning (decompensation) as well as intensive fibrotic hypertrophy (excessive thickening) led to an extreme increase in the risk of DU, but BWT in the physiological compensatory range led to protective effect (10, 28). This observed bi-directional morphological variance presents an intriguing hypothesis-generating nonlinear association. It suggests that complex structural remodeling might be overlooked by conventional linear statistical procedures, highlighting the need for prospective studies to physiologically validate this exploratory U-shaped relationship.

Moreover, our severe multidimensional feature selection algorithm (Boruta and RFE algorithm) clearly dismissed the conventional demographic and anatomical characteristics, especially patient age and total prostate volume (TPV). This observation is quite counterintuitive because age and size of the prostate are widely recognized as the leading progression factors of BPH (29). Nevertheless, this algorithmic exclusion aligns with the pathophysiological cascade of chronic bladder outlet obstruction. We hypothesize that downstream functional impairments (e.g., higher PVR, lower Qmax) and local structural remodelling (e.g., BWT and IPP) mediate (and consequently override) the predictive relevance of gross prostate enlargement and aging. By filtering out these upstream “surrogate” variables, the XGBoost model prioritized direct target-organ evidence, contributing to an efficient and interpretable non-invasive risk stratification framework.

The integration of local individualized SHAP explanations further bridged the translational gap between complex algorithmic outputs and practical clinical reasoning. Through detailed waterfall plots, we macroscopically quantified the sequential risk accumulation for specific patients. Importantly, the error analysis of a false-negative case highlighted the inherent limitations of non-invasive predictive models when confronting patients with paradoxical or deceptively preserved functional indices (30). In these borderline clinical cases, our results confirm once again that the use of additional invasive urodynamic tests is still essential to avoid such underdiagnosis and surgical failure (31).

Even though the diagnostic accuracy and interpretability of the method are promising, it has a number of limitations. First and foremost, this was a retrospective study conducted at a single tertiary institution. Therefore, there may be implicit selection bias in the study group, and the outstanding results of the XGBoost model could be partly explained by the homogeneity of the given population of patients. The generalizability of our risk stratification framework requires robust external and temporal validation through prospective, multicenter cohorts to confirm its sustained diagnostic stability. Second, the assessment of detrusor contractility relied on conventional urodynamic parameters, whereas advanced electromyographic or sophisticated pressure-flow analyses could potentially offer deeper mechanistic insights. Third, we based our model on assessment of anatomical features by mostly using transrectal ultrasonography, which, although very convenient, might not be as well characterized with high-resolution tissue phenotyping by multiparametric magnetic resonance imaging. Such prospective studies involving the combination of non-invasive DU phenotyping with extensive multi-omics data and functional imaging is justified in the future.

## 5 Conclusion

This study successfully developed and internally validated an interpretable XGBoost machine learning framework for the non-invasive prediction of detrusor underactivity (DU) in patients with benign prostatic hyperplasia. By rigorously reducing 15 conventional clinical parameters to a parsimonious five-feature subset, our model demonstrated superior discriminatory performance (AUC = 0.958) compared to traditional linear logistic regression. Crucially, the integration of SHAP analysis demystified the algorithmic “black box,” unveiling profound non-linear pathological trajectories—most notably, the U-shaped bi-directional risk profile of bladder wall thickness (BWT)—that evade standard univariable detection. Furthermore, local SHAP explanations effectively translated complex probabilistic outputs into individualized clinical reasoning. While these findings highlight the substantial potential of tree-based ensemble algorithms to optimize preoperative patient selection and minimize unnecessary surgical interventions, prospective validation across diverse, multicenter cohorts remains essential to ascertain the generalizability and real-world clinical utility of this predictive nomogram.

## Data Availability

The raw data supporting the conclusions of this article will be made available by the authors, without undue reservation.
