# Supplementary material for: Non-invasive prediction of detrusor underactivity in benign prostatic hyperplasia: an interpretable machine learning framework to optimize surgical selection
Source: Front Med (Lausanne). 2026 May 1;13:1835415. doi: 10.3389/fmed.2026.1835415 (PMC13175785; doi:10.3389/fmed.2026.1835415)
Supplement: Supplementary file 1 [file Table_1.docx]

**Supplementary Table S1. Baseline characteristics of the BPH patient cohort stratified by training and testing sets.**

| **Variable** | **Overall (n = 538)** | **Training Set (n = 378, 70.3%)** | **Testing Set (n = 160, 29.7%)** | **P-value** |
| --- | --- | --- | --- | --- |
| **Primary Outcome** |  |  |  |  |
| Detrusor underactivity (DU), n (%) | 243 (45.2%) | 171 (45.2%) | 72 (45.0%) | 1.000 |
| Non-DU, n (%) | 295 (54.8%) | 207 (54.8%) | 88 (55.0%) |  |
| **Demographics & Clinical History** |  |  |  |  |
| Age (years), Mean (SD) | 69.83 (8.73) | 69.80 (8.70) | 69.90 (8.80) | 0.895 |
| BMI ( kg/m ²), Mean (SD) | 24.04 (3.08) | 24.01 (3.09) | 24.11 (3.03) | 0.732 |
| Duration of symptoms (months), Median [IQR] | 38.00[24.00, 55.00] | 39.00 [24.00, 55.00] | 38.00 [22.75, 52.25] | 0.359 |
| **Laboratory Parameters** |  |  |  |  |
| Total PSA (ng/mL), Median [IQR] | 4.12 [3.53, 4.84] | 4.11 [3.52, 4.82] | 4.15[3.55, 4.86] | 0.682 |
| f/t PSA ratio, Mean (SD) | 0.21 (0.06) | 0.20 (0.06) | 0.21 (0.06) | 0.521 |
| **Ultrasonography & Urodynamic Parameters** |  |  |  |  |
| Total prostate volume (TPV, mL), Median [IQR] | 51.40[41.12, 60.77] | 51.30[40.70, 60.40] | 52.40[41.80, 61.28] | 0.177 |
| Transitional zone volume (TZV, mL), Median[IQR] | 25.20 [20.22, 30.58] | 25.10 [20.00, 30.50] | 25.40 [20.50, 30.70] | 0.651 |
| Transitional zone index (TZI), Mean (SD) | 0.50 (0.06) | 0.50 (0.06) | 0.51 (0.06) | 0.327 |
| Intravesical prostatic protrusion (IPP, mm), Median[IQR] | 7.20 [3.60, 11.30] | 7.25 [3.70, 11.57] | 6.80[3.18, 11.00] | 0.169 |
| Bladder wall thickness (BWT, mm), Mean (SD) | 4.54 (1.72) | 4.52 (1.71) | 4.58 (1.74) | 0.705 |
| Post-void residual (PVR, mL), Median [IQR] | 114.50 [67.00, 157.00] | 114.00 [64.25, 156.75] | 115.00 [73.75, 161.50] | 0.223 |
| Maximum flow rate (Qmax, mL/s), Median [IQR] | 8.85[5.90, 11.90] | 8.90[5.90, 12.20] | 8.75[5.88, 11.43] | 0.363 |
| **Symptom Scores** |  |  |  |  |
| IPSS-Voiding score, Mean (SD) | 12.55 (4.32) | 12.44 (4.40) | 12.82 (4.11) | 0.344 |
| IPSS-Storage score, Mean (SD) | 6.94 (2.78) | 6.96 (2.75) | 6.89 (2.85) | 0.791 |
| Quality of Life score (QoL), Mean (SD) | 4.03 (1.24) | 4.00 (1.23) | 4.11 (1.24) | 0.363 |

**Notes:**

Baseline characteristics are summarized for the overall cohort and stratified by the randomly assigned training and testing sets. Continuous variables are expressed as Mean (Standard Deviation) for normally distributed data, or Median [Interquartile Range] for non-normally distributed data. Categorical variables are reported as counts (percentages). P-values were calculated using Student’s t-test, the Mann-Whitney U test, or the Chi-square test, as appropriate. All P-values > 0.05 demonstrate no statistically significant difference between the two datasets, confirming a highly balanced data partitioning.

**Abbreviations:** BPH, Benign Prostatic Hyperplasia; DU, Detrusor Underactivity; BMI, Body Mass Index; PSA, Prostate-Specific Antigen; f/t PSA, free-to-total PSA; IPSS, International Prostate Symptom Score; SD, Standard Deviation; IQR, Interquartile Range.
